# Against methodological continuity and metaphysical knowledge

**DOI:** 10.1007/s13194-022-00505-6

**Published:** 2023-01-11

**Authors:** Simon Allzén

**Affiliations:** Philosophy Department, Stockholm University, Stockholm, Sweden

**Keywords:** Methodological continuity, Scientific Realism, Inference to the best explanation, Metaphysics

## Abstract

The main purpose of this paper is to refute the ‘methodological continuity’ argument supporting epistemic realism in metaphysics. This argument aims to show that scientific realists have to accept that metaphysics is as rationally justified as science given that they both employ inference to the best explanation, i.e. that metaphysics and science are methodologically continuous. I argue that the reasons given by scientific realists as to why inference to the best explanation (IBE) is reliable in science do not constitute a reason to believe that it is reliable in metaphysics. The justification of IBE in science and the justification of IBE in metaphysics are two distinct issues with only superficial similarities, and one cannot rely on one for the other. This becomes especially clear when one analyses the debate about the legitimacy of IBE that has taken place between realists and empiricists. The metaphysician seeking to piggyback on the realist defense of IBE in science by the methodological continuity argument presupposes that the defense is straightforwardly applicable to metaphysics. I will argue that it is, in fact, not. The favored defenses of IBE by scientific realists make extensive use of empirical considerations, predictive power and inductive evidence, all of which are paradigmatically absent in the metaphysical context. Furthermore, even if the realist would concede the methodological continuity argument, I argue that the metaphysician fails to offer any agreed upon conclusions resulting from its application in metaphysics.

## Introduction

Scientific realists think that we can have knowledge about unobservables entities such as electrons, atoms, and cells. One of the ways that realists have suggested we can gain this knowledge is via explanatory inference, or inference to the best explanation.^1^ If the existence of the unobservable entities features indispensably in science’s most empirically successful theories, we can justifiably believe in them, meaning that electrons, atoms, and cells are ontologically robust entities that we can have knowledge about. Most notably, this kind of realism has been explicated by Boyd (1983) and Musgrave (1988), and defended by Psillos (1999) and Kitcher (1995, 2001). Metaphysicians’ have argued that since they also employ inference to the best explanation, they are similarly justified in believing in the existence of abstract unobservable entities (Paul, 2012; Lyon, 2012; Colyvan, 2012; Swoyer, 1983; 1999; 2008). The metaphysicians’ gambit centers around methodological continuity: if scientific realists are justified in employing IBE to acquire knowledge about the unobservable parts of the world posited by science, metaphysicians who employ IBE must be similarly justified with respect to theories in metaphysics. The essential move in this strategy is to couple the defense of IBE in metaphysics to the success of the scientific realist endeavor to defend IBE in science against the anti-realist. I argue against this strategy.

My argument aims to show that the justification of IBE in science and the justification of IBE in metaphysics are two separate problems. The metaphysician needs these issues to be sufficiently similar in order for the methodological continuity argument to work. By tracking the dynamics of the debate regarding the justification of IBE in the literature on scientific realism, it becomes clear that any similarities between the two endeavors are superficial at best. I outline the dialectic in the debate on the justification of IBE with respect to scientific realism and evaluate the prospect of the metaphysicians’ ability to apply the realist arguments and strategies to the justification of IBE in metaphysics. As will become apparent, the metaphysician can, with the help of an unlikely ally, resist the separation of the issues at great length, but will ultimately have to abandon this strategy. The simple reason for this is that in the quest of convincing their empiricist antagonist, defenses of scientific realism have become increasingly empirical. The core issues in the debate on the justification of IBE in science has shifted from problems in the logical structure of arguments defending IBE to instead revolving around the constructive empiricist epistemic divide between observables and unobservables. The empiricist/realist debate on that divide is of course entirely orthogonal to the question of the reliability of IBE in metaphysics. In an additional argument I show that even under the supposition that the methodological continuity argument works, the metaphysician cannot plausibly argue that we have metaphysical knowledge, since there is no consensus among metaphysicians regarding which theories that provides the best explanations. That is, even if the scientific realist concedes that metaphysicians have a legitimate methodology, they seem unable to offer any cohesive results about what, exactly, we ought to be realists about. This lack of consensus suggests that the underlying problem in metaphysics is the absence of external methodological validation. Since there is no neutral, or additional, methodological vantage point from which to evaluate or assess the success of IBE in metaphysics, metaphysicians can only rely on a priori judgments to do so.

## Methodological continuity

The epistemic credentials of metaphysics has recently seen some heavy criticism. Ladyman and Ross writes that ’Standard analytic metaphysics [...] contributes nothing to human knowledge’. (2007, 1) Saatsi is less harsh but states that ‘[i]n the virtual absence of experiments, predictions, and empirical feedback, it is far from clear how metaphysical theories and views can be rationally justified’. (2017, 163) Attempting to defend the epistemic status of metaphysics from worries like the above, one naturalist strategy seeks vindication by invoking methodological similarity, the starting point of which is the observation that explanatory inferences are used in science and metaphysics. Here, Colyvan (2006) employs the methodological continuity argument as a justification for mathematical realism:

[I]nference to the best explanation is a special case of the indispensability argument. Moreover, as has already been noted, this is a style of argument that the scientific realist accepts. [...] So here I will take the indispensability argument to be an argument that puts pressure on the marriage of scientific realism and nominalism. It does this because the style of argument is one which scientific realists already endorse. (Colyvan, 2006, 227-8)

The last sentence makes an appeal to something like methodological similarity. Colyvan says that indispensability arguments (for mathematical realism) are vindicated by the fact that they are instances of IBE, a rule of inference that scientific realists accept. Another argument for methodological continuity comes from Swoyer (2008). Swoyer does not think that metaphysical explanations ‘are as deep or nuanced or successful as most explanations in chemistry or physics or physiology’, but he maintains that the connection between explanation and truth is sufficiently strong to propose that ‘Something similar can occur in philosophy.’ (2008, 17)^2^ Drawing on this alleged similarity of methodology his suggestion is that:

[w]e should (re)construe arguments for the existence of abstract entities as inferences to the best overall available ontological explanation. (Swoyer, 2008, 17)

Paul (2012) has given the argument its most developed defense, explicitly drawing on methodological continuity in defending the epistemic status of metaphysics:^3^

If [...] theoretical desiderata are truth conducive in science, they are also truth conducive in metaphysics (and in mathematics, and in other areas). The main point I want to make here is that if the method can lead us closer to the truth in science, it can lead us closer to the truth in metaphysics. [...] This is a central part of my thesis: if we accept inference to the best explanation in ordinary reasoning and in scientific theorizing, we should accept it in metaphysical theorizing. (Paul, 2012, 21-2)

The general argument metaphysicians have given for methodological continuity can be outlined as a modus ponens:

1. If IBE is truth-conducive in science, then it’s truth-conducive in metaphysics.
2. IBE is truth-conducive in science.
3. IBE in metaphysics is truth-conducive.

Paul argues that an upshot of the methodological continuity argument is that it forces a naturalistic scientific realist to endorse the view ‘that doing metaphysics, and philosophy more generally, is a rational and reasonable way to try to discover fundamental and general truths about the world.’ (Paul, 2012, 25) While the argument is logically valid, and builds on the fact that scientific realists accept (2) and the antecedent in (1), I will argue that realists have no reason to believe that (1) is true, since the reasons they have for believing in the antecedent are not reasons for believing in the consequent.

In applying the strategy of methodological continuity, the metaphysician relies on sharing the same fate as the realist with respect to the epistemic status of IBE – metaphysicians and realists succeed together, or fail together. This means that whatever argument, defense, gambit or strategy that realists utilize against their opponents, metaphysicians better hope that it applies, mutatis mutandis, to metaphysics as well. As we will see, this is not the case.

Here, we should give pause and reflect on the details of what the aim of the argument is. First, it is important to note that I do not deny the *possibility* that IBE is truth-conducive in metaphysics. I am not suggesting that there could never be an argument supporting IBE in metaphysics. What I am proposing, rather, is that the methodological continuity argument is dependent on a defense of IBE that works for science *and* metaphysics. The natural starting point of finding a defense of that kind is to look at the defenses which scientific realists have given already. The rationale for doing so is that the level of efficacy of the methodological continuity argument will be a function of the level of acceptance that the particular defense of IBE have among scientific realists. If metaphysicians can use the most favored defense of IBE in science in their methodological continuity argument, this would make life problematic for scientific realists. The main take-away point of my argument is that none of the favored scientific realist defenses of IBE already given extends to the justification of IBE in metaphysics via methodological continuity.

We may also reflect on how this argument differs from the two points of contention against methodological continuity presented by van Fraassen (2002, 13-5). His first point stresses the fact that explanations in science are evaluated much harsher than in metaphysics, and do not generally lead scientists to accept the best explanation as true, but rather *endorse* the best explanation. His second point is that the methodological continuity on offer is the use of IBE in science and in metaphysics, and that arguments against IBE in general undermine the argument. For scientific realists, none of these points appears attractive since they need IBE to be justified, *pace* van Fraassen’s second point, and in a stronger sense than mere theory endorsement, *pace* his first.

## Defending IBE

Many scientific realists believe that IBE can deliver the truth about unobservables posited in scientific theories. In other words, scientific realists believe that IBE is truth-conducive in science. What reasons do realists have for thinking that this is so? In the 1980’s, Boyd (1980, 1983) used a refined no-miracles argument (NMA) to defend scientific realism. Boyd’s argument is that the success of theory-driven scientific methodology is best explained by the truth of the theories which the methodology relies upon. Fine (1991) rejects the strategy of defending scientific realism by using IBE, claiming it to be question-begging and viciously circular:

[...] we can challenge whether any explanationist defense of realism is reasonable in the context of a debate over the reliability of the hypothetical method. For the issue under discussion in judging realism in this debate is precisely whether explanatory success provides grounds for belief in the truth of the explanatory story. To use explanatory success to ground belief in realism, as the explanationist defense does, is to employ the very type of argument whose cogency is the question under discussion. In this light the explanationist defense seems a paradigm case of begging the question, involving a circularity so small as to make its viciousness apparent. (Fine, 1991, 82)

According to Fine, the ‘ground-level’ IBE that is used in science cannot be defended by making an argument that depends on ‘meta-level’ IBE. Fine’s argument against realism is compatible with the metaphysicians’ strategy: if Fine is right, then realists cannot use Boyd’s version of NMA to defend IBE in science, and so cannot separate the issue from IBE in metaphysics. According to Fine, the realist must endorse her position on the very same basis as the metaphysician.

The debate over whether or not Fine is correct (and consequently whether or not (2) is true) is precisely the debate between scientific realists and empiricists over the reliability of IBE. A lot has happened in this debate since the early 90’s, where arguments for the reliability of IBE in science offered by the realist have been given in response to empiricist worries. It is unsurprising, then, that realists have tried to argue for the justification of IBE in ways that they hope would convince an empiricist. For example, Douven (2002) argues that a version of IBE which selects the best explanation from a set of known alternatives is immune to the ‘best of a bad lot’ argument from Van Fraassen (1990). While this fact – that the debate between scientific realists and empiricists has changed and fine-tuned realists preferred form of IBE – alone might seem worrying for the plausibility of the methodological continuity argument, I will review two of the most prominent approaches to defend IBE in science and assess whether the reasons given for the truth of (2) are also reasons to think that (1) is true.

### The explanationist defense of IBE

As stated above, Boyd (1980, 1983) provided several different versions of the no-miracles argument defending scientific realism which relied on IBE. Realism about scientific theories, Boyd claims, is the only scientifically plausible explanation for the instrumental reliability of scientific methodology. Boyd’s refinement of the NMA focuses on the empirical success of theory-driven scientific methodology. The best scientific explanation for this methodological success is, according to Boyd, scientific realism. The criticism from Fine stated that this particular way of defending IBE is viciously circular. Psillos (1999, 2007, 2009) mitigates the impact of this objection by distinguishing between premise-circularity and rule-circularity, and proceeds to develop the explanationist defense of IBE for scientific realism based on novel empirical success. If we suppose, in line with Psillos, that rule-circularity is benign, we may assess if the explanationist defense of IBE gives us reason to believe that (1) is true.^4^

A telling aspect of the explanationist defense of IBE that makes (1) implausible is that it takes the justification of IBE to be an a posteriori, empirically informed, process. The fact that scientific methodology is theory-laden and enjoys predictive and instrumental success is what gives us reason to believe that those theories we use in order to arrive at empirical success are (approximately) true. The relevant explanatory connection that the explanationist defense tries to establish is that between (novel) empirical success and truth. Only if the theory under consideration is empirically successful can one legitimately infer its truth. In this sense, the explanationist defense of IBE is dependent of the empirical success of scientific theories. It’s hard to imagine what (if any) metaphysical theory can be considered empirically successful, at least under any definition of empirical success given by scientific realists. For example, Psillos (1999, 105) claims that empirical success “should be more rigorous than simply getting the facts right, or telling a story that fits the facts”. Instead, the notion of empirical success that scientific realists use is one that “includes the generation of novel predictions that are in principle testable”.^5^ Given this definition, what in principle testable predictions do metaphysical theories generate? As a proof of concept regarding the lack of empirical success in metaphysical theories, we may use mereology: a field devoted to the relationship between a whole and its parts.^6^ In mereology, atomistic theories hold that “an atom (or “simple”) is an entity with no proper parts, regardless of whether it is point-like or has spatial (and/or temporal) extension”. Another set of theories in mereology claims that everything is made up of “atomless “gunk” [...] that divides forever into smaller and smaller parts”. Yet another suggests that “the whole cosmos is but one huge extended atom, an enormously complex but partless “blobject””. (Varzi, 2019). How are we to extract any useful in principle testable empirical predictions from these theories? Empirically successful contemporary theories in elementary particle physics like quantum field theory do not rely on any mereological assumptions for its success, nor can it be said to have any affinity with the “simples”, “gunk” and “blobjects” of mereology. Indeed, these three mereological theories seem to be underdetermined by data at the same time as they are mutually exclusive, suggesting that the chance that they could generate novel empirical predictions are slim to none.

The explanationist defense of IBE shows that the issue of justifying IBE in science is profoundly different from justifying IBE in metaphysics, and therefore that the consequent does not follow from the antecedent in (1). However, given that the explanatory defense of IBE itself relies on IBE, this defense is going to be dialectically inefficient against those who deny the legitimacy of IBE in the first place, i.e. empiricists. In an interesting turn of events, it would appear as if the metaphysician can utilize the empiricist arguments against the realist defense of IBE in so far as that particular defense is not also applicable to IBE in metaphysics. For the metaphysician, the enemy of their enemy is (sometimes) a friend. The metaphysicians’ strategy may then be, with respect to any realist defense of IBE, to first identify if the defense is applicable to IBE in metaphysics. If the realist defense of IBE is applicable to IBE in metaphysics, the methodological continuity argument is successful and metaphysicians can take part in a joint strategy with realists to argue for that particular defense of IBE against empiricists. If the realist defense is not applicable to IBE in metaphysics, the methodological continuity argument fails and metaphysicians can take part in a joint strategy with empiricists to reject that particular defense of IBE. Since the explanationist defense is not applicable to IBE in metaphysics, realists can refute the methodological continuity argument, forcing the metaphysician to side with the empiricist. Metaphysicians will then quickly point to the dialectical issue in the explanationist defense as the reason for not defending IBE in this particular way. Their reason for doing this would then be motivated by the fact that the explanationist defense, by virtue of its essentially empirical approach, is inapplicable to IBE in metaphysics.

### The inductive defense of IBE

Another realist strategy to defend the reliability of IBE in science is to invoke inductive evidence.^7^ According to this defense, we have reason to believe in the reliability of IBE in science because IBE has proven to be reliable in science in the past. This type of defense seeks to establish reliability by finding successful instances of explanatory reasoning and inference in the history of science. Again, this kind of defense has been developed in order to convince empiricists on empiricist terms. Bird (2006) offers an inductive approach which he hopes will convince the empiricist of the reliability of IBE with respect to the unobservables that realists regularly argue that we ought to believe in:

[Explanatory] inferences to the existence of unobservables have later been verified by direct observation once observational techniques have improved. We can now observe microbes and molecules, the existence of which was once a purely theoretical, explanatory hypothesis. (Bird, 2006, 160)

Bird’s inductive defense builds on the fact that we can confirm if past inferences in science were successful by later observation (or detection) of the inferred objects. This defense of IBE is, precisely as the explanationist defense, primarily focused on taking an empirical approach sufficient for purposes of convincing the empiricist. The empirical data is gained by verifying the explanatory inferences made in science by means of detecting the inferred objects.

Is Bird’s argument for IBE applicable to metaphysics? Given the empirical nature of the strategy and the scope of induction, it’s hard to see how it could be. If successful, Bird’s argument does not conclude that IBE is truth-conducive in the general way that is required for methodological continuity. The argument claims that explanatory inferences to unobservables in science are justified because explanatory inferences to unobservables in science have been empirically confirmed by detecting the inferred objects. The argument could even take a more local scope such that successful empirical confirmation of explanatory inferences in a specific scientific discipline justifies that inferential practice in only that discipline. Novick (2017) argues, building on Norton (2021), that this kind of local justification for inferences threatens methodological continuity:

If justification is local in this way, then the successful reliance on a theoretical virtue in a particular scientific context cannot support reliance on that virtue in metaphysics, unless it can be shown that the justification transfers across contexts. (Novick, 2017, 1172)

The metaphysician cannot assume that evidence for the reliability of IBE in one context is evidence for the reliability of IBE in any context. To be clear, it is certainly possible for this to be the case, but it has to be argued, not merely assumed. The burden of proof is on the metaphysician to demonstrate that the reliability of IBE is invariant with respect to its application in metaphysics or science. There are at least two ways for the metaphysician to proceed: the first is to find empirically confirmed successful applications of IBE in metaphysics (without assuming that they are successful – begging the question in precisely the way Fine worries about); the second is to show that the demonstrated justification of IBE is non-local to the scientific discipline in particular or to science in general.

As a means to show the former, the metaphysician can refer to the fact that the metaphysical theory of atomism developed by ancient Greek philosopher Democritus was confirmed empirically by the experiments of Perrin in 1908. While this case may carry some resemblance to an inductive defense of IBE, the claim that Democritus’ method was anything like IBE, more than merely a possible theory in logical space, is implausible. That Democritus’ theorizing was sufficiently similar to modern metaphysics is also highly implausible. Even worse, the two aspects of Democritus’ theory that were decidedly metaphysical both look questionable at best in light of modern physics. First, elementary particles in quantum field theory are not the eternal and indivisible ‘atoms’ of Democritus’ theory, but can be annihilated and transformed into different particles. Second, the vacuum of quantum field theory is a dynamical and fluctuating system, in no way resembling anything like Democritus’ empty space. That is, the only two aspects of the theory that were metaphysical in spirit, were precisely the aspects that subsequently became refuted.^8^ The empirical investigations that in all likelihood led Democritus to his conclusion – that most things can be divided into smaller things – are empirical facts that remain to this day. Even under the assumption that these worries can somehow be resolved, I suspect that metaphysicians would struggle to find enough cases to generate an induction. Bird’s strategy, then, decouples methodological justification in science from methodological justification in metaphysics by connecting justification with empirical evidence supporting an induction with limited scope.

In order for the methodological continuity argument to succeed, Bird’s defense must fail (or be supplemented). The metaphysician can again look to the empiricist for arguments against the inductive approach given by Bird. How is the scientific realist justified in saying that inferences to microbes and molecules have been subsequently confirmed by observation? The empiricist would not agree that the technology necessary to detect microbes and molecules is epistemically on par with observation – looking though a microscope is not an act of observing. This reflects the well known epistemic line drawn between observable and unobservable due to Van Fraassen (1980). The success of IBE in science cannot be checked by observation given the empiricist definition of the term. From the empiricist perspective, scientific realists are no better off than metaphysicians – both are going beyond the empirical evidence in making claims about the underlying structure of the observable phenomena. Metaphysicians can then claim that the empirical support for realism is a red herring simply because it never contains (observable) evidence of the reality of unobservable objects. Again, the empiricist has proven to be an unlikely ally against the realist refutation of methodological continuity, resulting in realists and metaphysicians still sitting in the same methodological boat. The dynamics in the dialectic are somewhat hard to follow, so it makes sense to reiterate what’s really going on. The metaphysician and the empiricist are unlikely allies only with respect to rejecting inherently realist defenses of IBE. The empiricist motivations for doing so is detached from the metaphysicians’ motivations: empiricists deny that we can know that theories dealing with unobservables are true, so it makes sense to reject an inference that promises to deliver precisely those truths; metaphysicians need the methodological continuity argument to work so must deny any defense of IBE that does not apply to metaphysics. Rejecting defenses of IBE sui generis to realism makes sense for the metaphysician because it means that the methodological continuity argument is still a live option. It is a live option since realists and metaphysicians will both be in a position of attempting to justify IBE, where the hope (at least for metaphysicians) is to find a more general defense, which would make the first premise in the methodological continuity argument true.

## The Galilean strategy

Perhaps realists can find what they need in Kitcher (2001), who offers another kind of inductive argument for the reliability of IBE. The idea behind Kitcher’s so-called Galilean Strategy is to justify a method M by testing it in an epistemic ‘environment’ already accepted by those skeptical about M. In Galileo’s case, this involved ensuring that the telescope delivered the truth about the celestial by demonstrating that it delivered the truth about the terrestrial, and that there was no principled distinction between them: induction over cases on earth established the reliability of the telescope even when making celestial observations. Kitcher argues that induction over success-to-truth inferences in the observable domain gets us similar methodological justification that we can then apply to the unobservable domain:

People find themselves in all sorts of everyday situations in which objects are temporarily inaccessible, or are inaccessible to only some of the parties. Detectives infer the identities of criminals by constructing predictively successful stories about the crime, bridge players make bold contracts by arriving at predictively successful views about the distribution of the cards, and in both instances the conclusions they reached can sometimes be verified subsequently. We readily envisage an idealized type of situation, perhaps most perfectly realized in some parlor games, in which the “success to truth” inference is tested and confirmed. (Kitcher, 2001, 176)

Kitcher suggests that when, in observational contexts where objects are temporarily unobservable, one entertains a host of theories some of them will prove to be successful and others not. At some later time, when objects are no longer temporarily unobservable, one will find out which theories were true and, according to Kitcher, also find a strong correlation between success and truth. The generalized argument is as follows:

1. Success-to-truth inferences are reliable in the observable domain.
2. We have no good reason to suppose that it will stop working in the unobservable domain.
3. Success-to-truth inferences are reliable in the unobservable domain.

The argument bears similarity to the methodological continuity argument, but importantly only argues for the reliability of success-to-truth inferences: the truth of a theory can be inferred only if the theory is empirically successful. Alas, as we saw earlier metaphysical theories are not empirically successful, at least not by any definition of empirical success acceptable to the realist. Ladyman (2012) makes a similar point:

[I]n so far as explanatory power is supported by its use in science and in everyday life it is coupled to empirical and practical success [...] We have inductive grounds for believing that pursuing simplicity and explanatory power in science will lead to empirical success, but no such grounds where we are dealing with distinctively metaphysical explanations, since the latter is completely decoupled from empirical success. (Ladyman, 2012, 46)

Much like the defenses above, the Galilean strategy takes the empirical approach inapplicable to the metaphysician, both in its formulation of the relevant inference as well as in its proposed confirmation. The question is whether or not the metaphysician can look to empiricism for help yet again.

### Reliability (dis)continued?

In a response to Kitcher, Magnus (2003) questions why the empiricist should have to accept that the lack of a defeater for the continued reliability of success-to-truth inferences from observable to unobservable entities provides reason to believe that this reliability holds. There is, in fact, no reason for empiricists to accept that it does.^9^ Empiricists may simply dispute that the truth of the second premise in Kitcher’s argument is enough to warrant the conclusion. Since we have no empirical evidence to support the use of IBE with respect to unobservables, there is no reason to suppose that IBE with respect to unobservables work. Lacking a reason to believe ¬*A* is not a reason to believe *A*.

It’s clear that Magnus’ objection cannot be used by the metaphysician in order to collapse the issues of justifying IBE in science and metaphysics. The first part of Kitcher’s argument, that IBE is reliable with respect to observables, is conceded by the empiricist. Magnus’ objection is premised on the possibility that the reliability of IBE is context-dependent, or local, and that realists need to provide evidence for its reliability in the relevant context, in their case the context of unobservables. The metaphysician is unable to utilise this empiricist objection because if they do, they have to concede that the reliability of IBE is, in fact, context-dependent, which undermines the whole point of the methodological continuity argument. We might, in order to conceptualise context-dependence, partition the levels of reality that each instance of IBE is proposed to be reliable with respect to. Empiricists are fine with IBE being reliable in the observable domain, scientific realists claim that reliability extends to unobservables indispensable for empirical success, and metaphysicians claim that reliability extends even further to include numbers, sets and relations (or abstracta, in general). A presupposition in the methodological continuity argument is that no such partition is relevant to the justification of IBE, which is precisely what is supposed to warrant the step from the antecedent to the consequent in the first premise. But as Magnus points out, this must be argued for, not merely presumed. The metaphysician is unable to echo the empiricist objection in order to collapse the issue of justifying IBE in science with justifying IBE in metaphysics, which ultimately renders the first premise in the methodological continuity argument false: even if IBE is truth-conducive in science, it does not follow that it is truth-conducive in metaphysics.

There are few attractive options left for metaphysicians seeking justification by methodological continuity, but one of them might be to join Magnus in his empiricist critique of the Galilean Strategy. One reason why they might want to do so, in spite the fact that it undermines methodological continuity, is that it levels the playing field by weakening the realist justification of IBE in science. The hope, even if slight, is that realists will be forced to abandon the Galilean Strategy at which point methodological continuity is a live option once more.

Realists now face a dilemma of sorts.^10^ Their first option, if they wish for their defense of IBE to be accepted, is to argue against Magnus. But this would require them to show that the reliability of IBE in one context is enough to show that IBE is reliable in general. If they succeed, then their own approach may be used by metaphysicians to argue from methodological continuity. The second option is to find empirical evidence for a local justification of IBE with respect to unobservables. This option, if successful, would not be subject to Magnus’ objection and would not be directly applicable to metaphysics. This is because the subject matter of much in metaphysical discourse is so far removed from the empirical context in which it would need to be tested that it seems to be a virtually impossible task for metaphysicians to solve. Realists are arguably in a better position. In the next section, I will examine an argument that seeks to explore the possibilities of justifying IBE locally with respect to unobservables in science.

## Defending inferences to unobservables

What could an empirical defense of IBE with respect to unobservables in science look like? In so far as the realist’s aim is to convince the empiricist while at the same time not overshooting the metaphysical implications, it would have to contain empirical evidence that empiricists find acceptable, meaning observable evidence. While it may sound as if realists searching for observational evidence of unobservables are conceptually confused, this depends on the relevant definition of unobservables. As we saw earlier, Bird argued that some entities that were considered unobservable became observable through technological advances. We also saw that on van Fraassen’s definition of observability, this was not acceptable. If the entities cannot be observed with the naked eye, they are not observable. If, however, there are cases where an entity has transitioned from being considered an unobservable (or theoretical) entity to being considered an observable (or empirical) entity, this should suffice to convince the empiricist about the legitimacy of IBE with respect to entities considered unobservable. This is so because in such cases realists have the means to evaluate the success of an inference to an entity considered unobservable that an empiricist accepts. This gives them empirical evidence in favor of inferences to entities considered unobservable. As a case study of what such a transition may look like, I will use Marie Curie’s discovery of radium.^11^

### Marie Curie’s inference to radium

In 1896, Henri Becquerel made a serendipitous discovery. He found out that uranium emanated a strange radiation the origin of which was internal to the substance itself. In light of the earlier exciting discovery of x-rays by Wilhelm Röntgen, Becquarel’s discovery, although relevant, was perceived as peripheral. It was Marie Curie who decided to investigate whether the somewhat underappreciated issue of uranium radiation was actually sui generis to uranium. She proceeded to test all the elements known at the time and discovered that in addition to uranium, thorium also radiated in the same way. Continuing her investigations, Curie decided to test several different chemical compounds of the elements and found out that the amount of radiation was invariant with respect to molecular structure. It did not matter if the uranium was in the form of a crystal or a powder – the radiation was constant. This led her to realize that radiation was a property of the structure of the atom, as opposed to the structure of the molecules. (Langevin-Joliot, 1998) The next step in her research was to analyze the mineral compounds, or ores, from which uranium and thorium were extracted. It was when she was doing this work that a puzzling result came about. The measured radiation from a pure uranium sample was significantly lower than the radiation from the compound mineral from which the uranium was extracted. Since her earlier results showed that the amount of radiation corresponds to the amount of uranium, this result is inexplicable. Marie Curie inferred that there must be an additional element with radiation properties present in the ore which must have been discarded in the process of extracting the uranium.

At this point, we may reflect on two points. The first is that the nature of the additional element is derived via theory and past experimental knowledge. It is Curie’s realization that radiation is an atomic property that rules out the possibility that the high levels of radiation could be due to some particular molecular structure in the ore. What this means is that the hypothesized element is theoretical at this point, meaning that its stipulation and nature is embedded in, and connected to, theory. The second is that Curie infers the existence of the entity based on the fact that it best explains the experimental results. Based on the experimental facts and background theory, she has drawn a conclusion based on explanatory considerations.

Proceeding in her research Curie decides to test her hypothesis. The standard process of testing any claims of discovering novel elements at the time was spectroscopic analysis. Every known element could be distinguished from each other by spectroscopic analysis because the elements reflected unique patterns of spectral lines in the machine. If Curie’s hypothesis was right, then a unique line associated with the new element would show up in the analysis. Spectrum specialist Eugène-Anatole Demarçay conducted the test and concluded that:

It does not seem possible to me that this line can be attributed to any known element [...] Neither barium nor lead from elsewhere [i.e. from sources other than the Curies’ material], as I have assured myself, give any line which coincides with it. (Demarçay, 1898, 175-178)

Strong evidence not only for Curie’s hypothesis, but also for the inference that led her to it. Alas, this evidence is not of the kind needed to persuade an empiricist. The inference is still to a theoretical, unobservable, entity. Despite this fact, the Curies took the evidence to imply the existence of radium:

The various reasons which we have just enumerated lead us to believe that the new radioactive substance contains a new element to which we propose to give the name radium. (Curie et al., 1898, 1216)

Curie had the same problem of persuading her contemporary colleagues in the scientific community. There would need to be hard evidence that radium actually existed. As a response to the demands from the scientific community, the Curies started the long and tedious process of isolating radium, an undertaking that would take several years and intense labor. In August 1902, after chemically processing 8 tons of the ore pitchblende, the Curies had managed to produce 1 decigram of pure radium chloride.^12^ The scientific result of this arduous process was that Curie’s hypothesis was proven right, but we may extract useful philosophical results from the process as well.

One of the philosophical results is that we have a case where an entity has gone from being considered a theoretical postulate to becoming an empirical quantity. The other philosophical result is methodological confirmation. The inference used by Curie gets empirical support from the fact that the inferred hypothesis was empirically proven by observational evidence. The question is if the inference should be construed as an inference to the unobservable or to the observable. It is clear that at the time the inference was made, the inferred entity was considered a theoretical postulate. It is also clear that the background theories provide no information about the observability of radium. It was possible, from a theoretical perspective, that radium was an extremely unstable and volatile element, so any attempt to isolate it would’ve failed. In this sense, and at that time, the inference was to an unobservable. However, this goes against the characterization of observability given by van Fraassen:

X is observable if there are circumstances which are such that, if X is present to us under those circumstances, then we observe it Van Fraassen(1980, 16)

Notice that the above characterization lacks a criterion for knowing if there are circumstances in which X is observable. It only says that if it is true that there are such circumstances, then X is observable whether we know it or not. An empiricist might then say that radium was observable all along simply because there were circumstances such that X was present to us in those circumstances, though this fact about the observability of radium was only known to us after Curie’s research had been made. It was a category mistake to consider radium unobservable or theoretical. Therefore, the discovery of radium does not lend itself to an empirical justification of inferences to unobservables. The focus of the debate has now shifted to the unobservable/observable distinction. In this debate, realists can of course ask if the distinction between the observable and the unobservable is really the salient epistemic divide that empiricists need it to be. Here, they might reiterate the points made by Churchland (1985) and Maxwell (1962). They may also question whether the distinction even makes any sense at all. It is certainly logically possible for all entities to be like Curie’s radium so that there always are circumstances in which an entity is observable to us, we are just ignorant about the particular circumstances. Our ignorance about the relevant circumstances may cause us to *consider* certain entities to be unobservable, but as Curie’s case shows we can certainly be wrong about such judgments. In such a situation, empiricists would be correct to say that we should limit epistemic commitment to observables, but it would be a trivial claim since many of the entities which in that case are observable would have been previously categorized as unobservable by the empiricist and therefore an unfit subject for rational belief. If so, the real question is whether we can have justified beliefs about entities prior to knowing that they are observable. The Curie case shows that we can.

We may now reflect on how the Curie case bears on the metaphysicians’ methodological continuity gambit. In light of Magnus objection to the Galilean Strategy, the metaphysician face two choices: i) accept that the Galilean Strategy justifies IBE in science but not in metaphysics, or ii) join the empiricist objection against the Galilean Strategy. The first option is undesirable for self-explanatory reasons, and the second one is undesirable because the core in the empiricist objection undermines the methodological continuity argument. Nevertheless, option (ii) would force the realist to either: (a) provide a reason to think that a local justification of IBE is sufficient for thinking that IBE is globally reliable, or; (b) provide a local justification for IBE with respect to entities considered unobservable. Since option (a) would support the reasoning in the methodological continuity argument, realists with nominalist inclinations would be wiser to choose option (b). The Curie case is an example of what option (b) could look like. It aims to establish that explanatory inferences made to entities considered theoretical or unobservable were confirmed empirically when those entities were discovered to be observable. While the debate about the epistemic merit of the observable/unobservable distinction has not yet settled, the important take-away point is that the debate about the justification of IBE in science is no longer an issue separated from the observable/unobservable issue, but very much a part of it. This is a debate in which metaphysicians have no stake. The kind of entities which metaphysicians trade in will not be able to become empirically detected, although it would certainly be worth reconsidering this point if sets and universals turned out to be distinguishable empirical quantities. The debate is no longer about the applicability of IBE, but about the observable/unobservable distinction and its epistemic significance for the empirical justification of IBE in science. Solving any problems related to that distinction is of no help to the metaphysician, since the issue of justifying IBE in metaphysics is orthogonal to it.

## Disagreement and metaphysical knowledge

Another argument against the prospects of the methodological continuity argument is that even if it is conceded, this does not mean that we have metaphysical knowledge. Suppose that you would ask scientists which scientific theories you should accept or what entities you should think are real. Most of them would give you a list consisting of roughly the same answers: atoms, genes, cells, planets, tectonic plates, general relativity, theory of evolution, chemical bonds, et.c. There is agreement among scientist with respect to many scientific theories. The process of scientific knowledge progresses by experiment, evidential analysis, theory revision, and unification. This process ultimately leads to a convergence of accepting a subset of scientific theories that have been well tested and confirmed. As a consequence, scientists are also in a position to evaluate the success of their employed explanatory reasoning in this process. In short, scientists mostly agree about the set of knowledge obtained from the scientific study of the world. Now suppose you would ask metaphysicians which metaphysical theories you should accept or what entities you should believe are real. Most of them would give you a unique list of their own favored theories – some would argue that you should believe in the theory of universals, others in trope theory; some would argue that the true theory of metaphysical composition is gunky, others would insist its funky, et.c. The process of gaining metaphysical knowledge does not proceed by experiment or evidential analysis and consequently metaphysicians do not converge on what theory that actually best explains some set of facts. This discrepancy reveals two rather striking ways in which science is essentially different from metaphysics with respect to providing knowledge about the world: i) while scientists do employ IBE, it is only one of many different epistemic dimensions in science. While IBE matters in the process of gaining scientific knowledge, it is not sufficient to do so. This is clear from the fact that even though particle physicists had trust in the Higgs hypothesis in virtue of its explanatory power, they still built the largest, most expensive, most complex machine in the history of humanity to test it.^13^ ii) the fact that science can pursue a plurality of methodologies means that it can update and fine-tune the epistemic details of each method. In the case of IBE, scientists can assess the explanatory virtues they use by checking how well they perform when subsequently testing the theories experimentally. This is a non-starter for metaphysicians. Remarkably, Paul considers the lack of convergence in metaphysics a virtue:

In ontology, because of the large size of the class of empirically adequate competitors, it is rare to have the application of theoretical desiderata winnow down the field to a single theory. There are usually a number of remaining competitors, each of which exhibit some combination of theoretical virtues combined with varying ways of accommodating the basic characteristics that are supposed to compose the empirical data. A bonus of this situation, not to be underestimated, is the value of epistemic diversity or disagreement: having different acceptable theories in competition with each other can contribute to the depth and quality of our overall ontological account of the world. (Paul, 2012, 22)

Paul is arguing that epistemic diversity, or epistemic disagreement, possibly contributes positively to depth and quality of ontology, but fails to explain how, other than hinting at the idea that theoretical rivalry necessarily leads to better theories. It is unclear how we are supposed to evaluate which theory is better if they are all still empirically underdetermined exhibiting some combination of theoretical virtues. I would argue that this disagreement undercuts, rather than supports, the idea of epistemic realism in metaphysics that Paul is advocating:

The metaphysical realist’s theory of the fundamental natures of the world is indirectly confirmed by its success as a theory that fits with ordinary experience and by how well it fits with other well-accepted theories, including empirically confirmed scientific theories. (Paul, 2012, 19)

If indirect confirmation via fit is to have any epistemic leverage at all, it must *not* be the case that a substantial number of metaphysical theories all fit ordinary experience, well-confirmed scientific theory, and display some combination of theoretical virtues. But that is precisely what disagreement shows is the case. If we are supposed to be metaphysical realists because metaphysicians use methodology warranted in science, then this methodology had better produce convergence with respect to the metaphysics we are supposed to be realist about. The lack of convergence among metaphysicians shows that even if the methodological continuity argument succeeds, there would be no agreed upon set of metaphysical knowledge on offer when asked what we should be realists about. It also shows that the underlying problem in metaphysics is lack of external methodological validation. There is no neutral, or additional, methodological vantage point from which to evaluate or assess the success of IBE in metaphysics. This fact explains the lack of convergence. The challenge for the metaphysician, in order to convince scientific realists that there is metaphysical knowledge, is to show that the following argument is true: If IBE is truth-conducive in metaphysics, and if metaphysicians converge on theories using IBE, then there is metaphysical knowledge. In what I have argued, scientific realists have reason to think that neither conjunct is true.

## Summary

I have argued that the scientific realist has good reason to discard the metaphysicians argument from methodological continuity. I argued that the metaphysician seeking to piggyback on the realist defense of IBE in science by invoking methodological continuity presupposed that the defense is straightforwardly applicable to metaphysics. As we have seen, it is not. The favored defenses of IBE in scientific realism make extensive use of empirical considerations, predictive power and inductive evidence, all of which are paradigmatically absent in the metaphysical context. While the metaphysician is able to use some empiricist objections to refute empirical defenses of IBE, not all such objections can be used to collapse the issues of justifying IBE in science with justifying IBE in metaphysics. Particularly troublesome was Magnus’s objection which, if accepted, undermined the methodological continuity argument. I also explored a case study of Curie’s inference to the existence of radium as a possible way for realists to counter Magnus’s objection without inadvertently supporting methodological continuity. The case turned on the epistemic salience of the observable/unobservable distinction by van Fraassen. Solving problems related to that distinction is of no help to the metaphysicians’ methodological continuity argument, since the issue of justifying IBE in metaphysics is orthogonal to the distinction.

Furthermore, I argued that the metaphysician, even if the realist would concede the methodological continuity argument, failed to offer any agreed-upon conclusions resulting from its application in metaphysics. The fact that metaphysicians disagree about which metaphysical theory provides the best explanation shows that, even when granted a sound methodology, its application in metaphysics has been unsuccessfully executed.

## Footnotes

1 The most comprehensive defense of IBE is made by Lipton (2003).

2 See also Swoyer (1983, 1999) for arguments concerning why metaphysicians ought to draw on methodological continuity.

3 Brenner (2017) defends a modest version of this claim, focusing on simplicity. While simplicity is sometimes thought of as one of the guiding virtues of IBE, it is more useful to focus on IBE in general, setting aside the question about the specifics of theoretical virtues.

4 From an empiricist point of view, one may not so easily concede that rule-circularity is benign, but for the present purposes, the question of viciousness is orthogonal. For an evaluation of rule-circularity and IBE, see Carter and Pritchard (2017).

5 For another definition of empirical success, see Kitcher (1995).

6 The take-away point here is that much of what goes on in metaphysics is decoupled from empirical matters, making it decoupled from realist defenses of IBE.

7 See Douven (2002) and Harré (1986) for inductive approaches to defend IBE.

8 Should anyone object to the jump from Democritus to Perrin to quantum field theory, I encourage them to reconstruct the history of atomism in its entirety. This may generate a stronger case for methodological continuity, or it may not. The burden of proof lies on the metaphysician to show the former.

9 Despite Kitcher referring to such empiricist denial as ‘the height of metaphysical hubris’.

10 It may worthwhile to point out that metaphysicians also face a dilemma or sorts. To argue against the various defenses of IBE given by realists, they may be taken to undermine, or erode, the plausibility of IBE in general. If so, the dilemma is whether to undermine IBE in general, including metaphysics, or to say that the realist defenses are sound, but admit that they are not applicable in metaphysics. Thanks to an anonymous referee for pointing this out.

11 For a detailed version of this argument, see Allzén (2022).

12 Marie Curie and André Debierne would some years later also manage to isolate radium in its metallic form from a solution of radium chloride using electrolysis (Ropp, 2012).

13 For an excellent analysis of this trust see Dawid (2017).
